# Global and local interference effects in ensemble encoding are best explained by interactions between summary representations of the mean and the range

**DOI:** 10.3758/s13414-020-02224-7

**Published:** 2021-01-27

**Authors:** Marco A. Sama, Dilakshan Srikanthan, Adrian Nestor, Jonathan S. Cant

**Affiliations:** 1 Department of Psychology, University of Toronto Scarborough, 1265 Military Trail, Toronto, Ontario M1C1A4 Canada; 2 Department of Laboratory Medicine and Pathobiology, University of Toronto, Toronto, Ontario Canada

**Keywords:** Ensemble encoding, Global-local processing, Interference, Visual working memory

## Abstract

Through ensemble encoding, the visual system compresses redundant statistical properties from multiple items into a single summary metric (e.g., average size). Numerous studies have shown that global summary information is extracted quickly, does not require access to single-item representations, and often interferes with reports of single items from the set. Yet a thorough understanding of ensemble processing would benefit from a more extensive investigation at the local level. Thus, the purpose of this study was to provide a more critical inspection of global-local processing in ensemble perception. Taking inspiration from Navon (*Cognitive Psychology, 9*(3), 353-383, 1977), we employed a novel paradigm that independently manipulates the degree of interference at the global (mean) or local (single item) level of the ensemble. Initial results were consistent with reciprocal interference between global and local ensemble processing. However, further testing revealed that local interference effects were better explained by interference from another summary statistic, the range of the set. Furthermore, participants were unable to disambiguate single items from the ensemble display from other items that were within the ensemble range but, critically, were not actually present in the ensemble. Thus, it appears that local item values are likely inferred based on their relationship to higher-order summary statistics such as the range and the mean. These results conflict with claims that local information is captured alongside global information in summary representations. In such studies, successful identification of set members was not compared with misidentification of items within the range, but which were nevertheless not presented within the set.

## Introduction

Ensemble encoding is a well-established phenomenon where the visual system compresses redundant statistical properties from a given set of stimuli into a summary metric at the expense of detailed representations of single items. Ariely’s (2001) classic study demonstrated this, revealing that participants could accurately report the mean size of a set of circles, but were poor at identifying the size of any one individual circle from the set. Despite these findings, people typically have a subjective impression of seeing the entire stimulus set in good detail (Cohen, Dennett, & Kanwisher, 2016; Yamanashi Leib, Kosovicheva, & Whitney, 2016). In reality, the limited capacity of our visual working memory (VWM) places restrictions on the level of detail that we can extract from individual items within an ensemble. This disconnect between our subjective impression and veridical perceptual processing has been an enduring interest of cognitive scientists for decades.

Typical statistical representations include the mean (Corbett & Melcher, 2014; de Fockert & Wolfenstein, 2009; Haberman & Whitney, 2009; Utochkin, 2015) and variance (Haberman, Lee, & Whitney, 2015; Suárez-Pinilla, Seth, & Roseboom, 2018) of an ensemble. This parallels gist-based processing found in scenes (Brady, Shafer-Skelton, & Alvarez, 2017; Campana, Rebollo, Urai, Wyart, & Tallon-Baudry, 2016; De Cesarei & Loftus, 2011; Greene & Oliva, 2009; Hedgé, 2008), which are represented by a number of features that capture the statistical regularities of the visual world. This includes, but is not limited to, open versus closed spatial boundaries and mean depth (Greene & Oliva, 2009; Park, Brady, Greene, & Oliva, 2011). Scene categorization occurs quite quickly, within 150 ms or faster (Fabre-Thorpe, 2011; Li, VanRullen, Koch, & Perona, 2002; Peelen, Fei-Fei, & Kastner, 2009; Thorpe, Gegenfurtner, Fabre-Thorpe, & Bülthoff, 2001). In addition to basic categorization, these fast scene-processing mechanisms also include the processing of global spatial properties, such as recognizing a scene as natural or urban. Interestingly, there are common neuroanatomical substrates mediating both scene and object ensemble processing (Cant & Xu, 2012, 2015, 2017, 2020).

Numerous studies have revealed that summary statistical extraction takes place at various levels of stimulus complexity, from simple shapes to facial identity (for reviews see Alvarez, 2011; Srinivasan, 2017; Whitney & Yamanashi Leib, 2018). Elucidating the underlying cognitive mechanisms of this process has received increased attention in recent years, shifting the focus from “what” is extracted to “how” it is extracted. This research has revealed that ensemble processing mechanisms operate independently from traditional single-item processing (Cant, Sun, & Xu, 2015; Im & Halberda, 2013) as well as across higher- and lower-level stimulus domains (Haberman, Brady, & Alvarez, 2015; Sama, Nestor, & Cant, 2019). Furthermore, ensemble perception appears to require minimal levels of attention (Chong & Treisman, 2005; Ji, Rossi, & Pourtois, 2018; Khayat & Hochstein, 2018; Peng, Kuang, & Hu, 2019; Utochkin & Tiurina, 2014; but see Jackson-Nielsen, Cohen, & Pitts, 2017). Like gist information from scenes, ensemble information is extracted quickly, as fast as 50 ms, with little change for exposure times up to 1,000 ms (Chong & Treisman, 2003; but see Whiting & Oriet, 2011, for a more conservative estimate).

Many previous studies of ensemble processing have focused disproportionately on extraction of visual information at the global level. This necessitates a more robust understanding of the mechanisms that operate at the local level of ensemble representation. Here, we highlight two important and related questions. First, what is the nature of single-item representations in the context of ensembles? Some studies suggest individual item values are captured (Li et al., 2016; Neumann, Ng, Rhodes, & Palermo, 2018), while others disagree (Chong & Treisman, 2003; Corbett & Oriet, 2011; Ward, Bear, & Scholl, 2016). Importantly, many of these ensemble studies use stimuli that are quite homogenous, which makes it difficult to disambiguate values of individual items. For example, when using circular stimuli, Corbett and Oriet (2011) varied individual exemplars by 4 pixels, and Chong and Treisman (2003) varied them by less than 1° of visual angle. A more comprehensive investigation is required using items with wider variability to enhance discriminability.

Second, what is the relationship between the processing of global summary statistics and the details of local items? Studies show that single-item reports tend to be biased to the ensemble mean (Brady & Alvarez, 2011; de Fockert & Wolfenstein, 2009; Maule, Witzel, & Franklin, 2014; Sama et al., 2019). The reverse may also occur, wherein focused attention on single items biases reports of the ensemble mean (de Fockert & Marchant, 2008), either through primacy and recency effects during serial presentation (Hubert-Wallander & Boynton, 2015), or if certain single items are intrinsically valuable (Dodgson & Raymond, 2019). In these situations, select single items become more salient (Kanaya, Hayashi, & Whitney, 2018), and are thus more likely to be captured and remembered. This type of pop-out is dissimilar from an outlier effect (Cant & Xu, 2020; Hochstein, Pavlovskaya, Bonneh, & Soroker, 2018). For example, the influence of outliers can be discounted from the overall summary representation (Haberman & Whitney, 2010), whereas enhanced salience of single items, which are not considered outliers, are included in summary representations and can bias the perception of the mean more than the other items.

Whether single-item interference on global summary processing can occur in the absence of salience at the local level awaits clarification. Until then, this remains an important avenue of investigation as a clearer understanding of the global-local relationship will inform models of ensemble processing. For example, the presence of local interference would suggest that prototypical exemplars may bias representations of global averages. Additionally, the existence of a mechanism mediating local interference would provide a new opportunity to investigate the current subsampling debate that concerns the number of items sampled when calculating a summary statistic (Bauer, 2015; Chong, Joo, Emmanouil, & Treisman, 2008; Lau & Brady, 2018; Maule & Franklin, 2016; Myczek & Simons, 2008). With this in mind, in the present study we generated an ensemble paradigm that could evaluate bidirectional interference across global and local levels of ensemble processing.

The most influential study of global-local processing arguably comes from Navon’s (1977) seminal work. Here, compound letter stimuli consisting of a larger letter made up of smaller letters were presented to participants. Global-local properties could be consistent (e.g., a large letter *H* made up of smaller *H*s) or inconsistent (e.g., a large letter *S* made up of smaller letter *H*s). Participants were tasked with reporting either the global or local letter across varying consistency. Participants tended to be slower and less accurate when reporting the local compared with the global properties of the compound stimuli. This was further reduced when the global configuration was inconsistent with the local letters. These now classic results were termed the global precedence and global interference effects, respectively. Taken together, compound stimuli, along with scenes and ensembles, are all composed of global and local structures. They each benefit from global statistical processing, which in turn influences local processing. Interestingly, research has also shown local interference for compound stimuli (Rijpkema, van Aalderen, Schwarzbach, & Verstraten, 2007) and scenes (Lowe, Ferber, & Cant, 2015). Given the functional relationship between scenes and ensembles (Cant & Xu, 2012), it is reasonable to predict that some local interference may occur in ensemble processing, even in the absence of salience at the local level.

With inspiration from Navon (1977), we designed a novel ensemble paradigm that could independently manipulate levels of interference at either the global or the local level. Our stimuli were not designed to look like classic Navon stimuli, but instead the paradigm we designed was inspired by Navon’s work. That is, participants make discriminations of global and local features of an ensemble stimulus in the presence of independent manipulations to the level of interference (with low interference loosely relating to consistent trials in Navon’s terminology, and high interference loosely relating to inconsistent trials). Participants were shown ensembles consisting of eight isosceles triangles, each with a clearly defined orientation direction. This allowed ensembles to have a mean angle spanning all 360°. Following the ensemble display, participants were asked to report either the set’s average orientation, or the orientation of a randomly selected single triangle. We used a two-alternative forced choice (2AFC) task, consisting of the correct target, which could be either the mean of the set or the true orientation value of the single triangle depending on the task, and an incorrect distractor. The degree of interference from the distractor was manipulated, allowing us to measure the influence of high versus low interference on reporting the target. In Experiment 1, when reporting average orientation, the high interference distractor was a single triangle from the set, and the low interference distractor was a single triangle not presented within the set. When reporting the orientation of a single triangle, the high interference distractor was the average orientation of the set, and the low interference distractor was a single item not from the set. Thus, high interference distractors were always a property of the ensemble set, coming from the opposing hierarchical level based on the participant’s task. Low interference distractors were values not contained within the ensemble set. Importantly, the average orientation was never explicitly presented within any ensemble display in any Experiment.

Given the parallel to Navon’s (1977) study when designing our novel ensemble paradigm, we expect to see analogous findings. Specifically, the global precedence effect would manifest as faster reaction time (RT) and improved accuracy when reporting the average orientation compared with a single orientation. When reporting single-item orientation, global interference would occur if participants were more likely to select the high-interference distractor, misidentifying the mean of the set for the correct single orientation. We also predict that local interference will be observed, which would occur if participants selected a high-interference distractor when reporting average orientation, mistakenly identifying a single item value for the mean orientation.

To our knowledge, this study is the first to reveal the parallels between ensemble processing and Navon-style global precedence and global interference^1^ effects. Importantly, the results from our first exploratory experiment informed the design of five subsequent experiments concerning the nature of single-item representation and the potentially reciprocal influence of global and local processing in ensemble encoding. Over six experiments, we show that what may initially appear as reciprocal interference is actually the result of a relationship between two summary attributes of the ensemble – the mean and the range.

## General methods

### Participants

Recruitment took place at the University of Toronto Scarborough. The university ethics board approved the study. Compensation was monetary or with course credit. Participants all provided informed consent prior starting the experiment, had normal or corrected-to-normal vision, and had no history of neurological impairment. We also restricted eligibility to right-handed participants. In total, we conducted six experiments. The final sample sizes of each experiment were as follows: Experiment 1 had 25 participants (ten males, age range: 18–29 years), Experiment 2 had 24 participants (ten males, age range: 18–23 years), Experiment 3 had 29 participants (six males, age range: 18–21 years), Experiment 4 had 24 participants (nine males, age range: 18–21 years), Experiment 5 had 26 participants (six males, age range: 18–26 years), and Experiment 6 had 24 participants (ten males, age range: 18–29 years).

### Stimuli and apparatus

Experiments were programmed with MATLAB version 2016 (https://www.mathworks.com) using the psychophysics toolbox (Brainard, 1997). The experiment took place in a darkened room using a desktop computer running Windows 10. Stimuli were displayed on a 60-Hz 24-in. LCD monitor with a 1,920 × 1,080 pixel resolution.

Participants sat with their eyes positioned 60 cm from the screen, held in place with the aid of a chinrest. Ensemble stimuli consisted of eight blue isosceles triangles presented on a black background. The base and height of each stimulus was 25 and 100 pixels, respectively. To ensure good contrast from the black background, we added a white border around each stimulus. Triangle stimuli subtended a visual angle of 0.6° for the base and 2.4° for the height. An entire ensemble display subtended a visual angle of 14°.

**Fig. 1:**
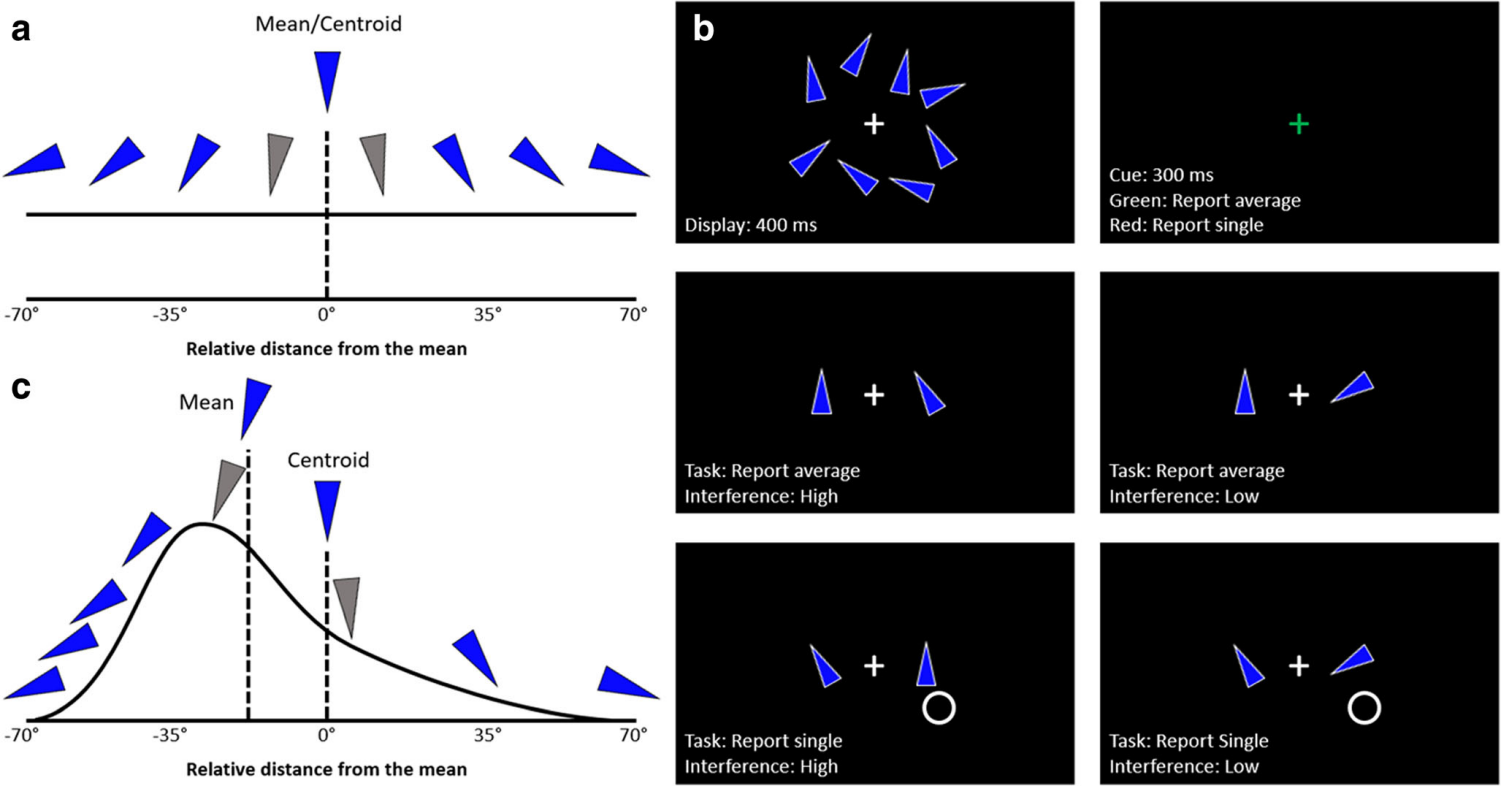
Trial and stimuli design. (**A**) Visualization of the uniform ensemble distribution used in most experiments. Each triangle orientation varies in 20° increments, with a range of 140°. (**B**) A sample ensemble display (top left) with a mean of 180°. The interstimulus interval (ISI; top right) followed the display, where the fixation cross cued participants to the task (green = report average; red = report single). After the ISI, participants responded via the use of a 2AFC task, which presented a correct target (left item in lower four panes) and an incorrect distractor (right item in lower four panes). Note, the spatial presentation of target and distractor items was randomized on each trial in each experiment. Unknown to participants, the extent of interference carried by the distractor was manipulated in each experiment (except for Experiment 3, which did not use a 2AFC design). During report single (lower two panes), a white circle was presented to indicate the location of the target single item. Note that these displays are not visually to scale and did not contain the descriptive white text. (**C**) Positively skewed ensemble distribution used in Experiment 6, showing relative placement of the mean and centroid values. Grayed items (**A** and **C**) were omitted as potential targets or distractors, as they are too close on the orientation continuum to their respective mean. They were still displayed (in blue) in the ensemble display

The mean ensemble orientation on each trial was randomly selected from a set of predetermined mean values, which varied across experiments (see *Methods* of each experiment for specific details). The orientation values of the eight individual triangles were then generated around this mean. To reduce homogeneity and ensure each item could be individuated from its neighbor, we used wide incremental steps between adjacent triangles on the stimulus continuum. Specifically, for all Experiments (except Experiment 6, which modified distribution skew), the orientations of the individual triangles were -70°, -50°, -30°, -10°, +10°, +30°, +50°, and +70° from the mean value of a given ensemble (Fig. 1A displays this uniform distribution).

Triangle stimuli were displayed surrounding a central fixation cross in a clocklike arrangement, angled 45° relative to the center fixation cross to spread them evenly. Half the items had a radius of 200 pixels from the center, and the other half had a radius of 300 pixels, and these selections were chosen randomly. We did this to prevent any illusory holistic structure that may emerge if the items were displayed along a perfect ring. Figure 1B (upper left pane) illustrates a sample ensemble display. Note the stimulus scale has been enlarged to aid visibility.

### Procedure

The general procedures for all experiments are as follows: participants first provided informed consent, completed a basic demographic questionnaire, were given instructions on the experimental task, and finally completed practice and experimental trials. Feedback was given after each practice trial, ensuring participants understood the task, but was omitted during the experimental trials.

Figure 1B illustrates the general phases of a trial. Note the general layout was different for Experiment 3, which used a different experimental task altogether. Each trial began with a white fixation cross, displayed at the center of the screen for a variable 400- to 1,000-ms duration. Participants were instructed to maintain fixation. Next, an ensemble was displayed for 400 ms (Fig. 1B, upper left pane). The ensemble display was then removed from the screen and was followed by a 300-ms interstimulus interval (ISI; Fig. 1B, upper right pane). The fixation cross remained onscreen during this phase, with an altered color to reflect the participants’ task (this did not occur in Experiment 3). The presence of a green cross cued participants to report the average orientation value (Fig. 1B, center two panes), whereas a red cross cued participants to report a randomly selected single item value, indicated by the presence of a white circle where that single item was previously displayed (Fig. 1B, lowest two panes).

Finally, after the ISI, two probes were presented to the left and right of the central fixation cross during the 2AFC task (Fig. 1B, lower four panes). The placement of the distractor and target probe were randomized in each trial, and the probes remained onscreen until a response was made. Using the number bar, participants pressed “1” on the keyboard to select the left probe, or “2” to select the right probe. This phase differed in Experiment 3, where only one probe was presented and participants were tasked with judging its set membership using “1” to identify the probe as a member of the preceding set and “2” as not a member of the set. In all experiments, participants were told to make their responses as quickly and accurately as possible.

The level of interference from the distractor probe was manipulated differently in each Experiment (see specific experiment *Methods* sections), which was unknown to participants. Typically, a high interfering distractor during report average would be set to the value of one of the single items from the set. Likewise, a high interfering distractor during report single would be set to the mean value. Conversely, low interfering distractors typically contained values outside the range of the ensemble (but refer to *Methods* sections from specific experiments to see exceptions to these rules).

### Data analysis

The combination of task and level of interference gives four unique conditions: report average high interference, report average low interference, report single high interference, and report single low interference. For each participant separately, outlier analyses removed trials where RT surpassed ± 2.5 *SD* from the mean of each unique condition. We judged trials with an RT faster than 150 ms to be an anticipatory response and removed them. For every condition, mean RT was calculated from the remaining trials (both correct and incorrect trials were used, based on the expectation that there would be significantly different numbers of trials across the report average and report single tasks, owing to the predicted accuracy differences across tasks), and accuracy was calculated as a percentage of correct responses. During group analysis, an additional outlier analysis removed participants whose data fell outside the 2.5 *SD* range for either RT or accuracy. Participants were also excluded for poor performance if more than ten trials for any given condition were removed. This was an appropriate criterion as most participants had less than six trials removed per condition.

Most experiments utilized a 2 (Task: report average or report single) × 2 (Interference: high or low) repeated-measures ANOVA, analyzing RT and accuracy separately. We report Greenhouse-Geisser corrected *p* values and modified *df* whenever sphericity was violated. Post hoc pairwise *t*-tests to investigate significant main effects or interactions were all two-tailed, whereas one-tailed *t*-tests were used to compare the accuracy of a given condition to chance levels of performance (i.e., 50%). Effect sizes for statistically significant findings are reported with partial *η*^2^ or Cohen’s *d* where applicable. Multiple comparisons were corrected for using the Bonferroni procedure.

## Experiment 1

To evaluate single-item representation in ensemble perception, and their possible relationship with global summary statistics, we investigated whether processing single items can interfere with reports of average orientation. If local interference occurs, we expect this to manifest as decreased accuracy and possibly increased RT when reporting average orientation under high local interference. Moreover, our ensemble interference paradigm allows us to investigate parallels between ensemble perception and the classic global precedence and interference effects (Navon, 1977). Global precedence would manifest as faster RT and increased accuracy for the global conditions compared with the local conditions. For global interference, we expect to see performance detriments on accuracy and RT when reporting local orientation under high global interference.

### Stimuli and procedures

The ensemble stimuli and general trial sequence was described in the *General methods* (Fig. 1). In brief, a trial began with a variable 400- to 1,000-ms fixation period, followed by the presentation of the ensemble consisting of eight blue triangles for 400 ms, then a 300-ms ISI where the fixation cross changed color to cue the participant to the task (green = report average orientation; red = report single orientation) in the ensuing 2AFC phase (which remained onscreen until the participant’s response). There were 15 possible ensemble means, evenly spaced from 0°, giving one possible mean angle every 24°.

Unknown to participants, the level of interference was manipulated for reports of average and single orientation. In high-interference trials, the distractor was a property of the ensemble: the orientation of a randomly selected single item from the set when participants reported the average, or the average orientation when participants reported the orientation of a single triangle from the set. In low-interference trials, the distractor was an orientation value that was not included in the preceding ensemble, and instead was 160–180° from the ensemble mean (90–110° outside the range). The interference manipulation coupled with the two tasks yielded four unique conditions: average high-interference, average low-interference, single high-interference, and single low-interference (Fig. 1B, lower four panes). This design is analogous to traditional global-local processing paradigms where both hierarchical levels could be either consistent or inconsistent with one another. Each of the 15 predetermined means was repeated six times across the four conditions, giving 360 total trials.

### Results

**Fig. 2:**
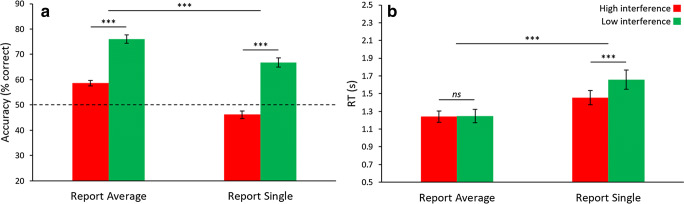
Results of Experiment 1. (**A**) Accuracy displayed patterns consistent with global precedence and global interference. Participants were more accurate during report average and during low interference. When reporting single under high interference, they typically mistook the mean as the correct single item. (**B**) Reaction time (RT) results were similar to accuracy, in that participants were faster for report average. One difference from accuracy was the lack of significance between the two levels of interference during report average. Error bars represent ± 1 standard error. *** *p* < .001, *ns* = not significant (*p* > .05)

Accuracy and RT for Experiment 1 were analyzed separately, each with a 2 (Task: report average or report single) × 2 (Interference: high or low) within-subjects ANOVA (the same results were found when restricting the RT analysis to correct trials only, so here, and moving forward, all RT analyses focus on both correct and incorrect trials). For accuracy (Fig. 2A), we report significance of Task (*F*_1,24_ = 92.06, *p* < .001, *η*^2^ = .793) and Interference (*F*_1,24_ = 93.56, *p* < .001, *η*^2^ = .796), but a non-significant Task-Interference interaction (*F*_1,24_ = 1.77, *p* = .195). Post hoc pairwise testing revealed accuracy was lower under high interference compared with low interference during reports of both average orientation (mean difference = 17.4%, *t*_24_ = 10.95, *p* < .001, *d* = 2.24) and single orientation (mean difference = 20.6%, *t*_24_= 7.25, *p* < .001, *d* = 1.48). Performance for all four conditions was significantly different from chance (all *t*s > 2.53, all *p*s < .037 after Bonferroni correction, all *d*s between 0.51 and 3.06). Interestingly, report single high interference was the only condition where performance was significantly below chance, indicating that participants were more likely to select the mean orientation, which was not present in single items making up the ensemble, over the correct single item. These results are consistent with previous ensemble research (Khayat & Hochstein, 2018, 2019).

For RT (Fig. 2B), Task (*F*_1,24_ = 67.68, *p* < .001, *η*^2^ = .738) and Interference (*F*_1,24_ = 11.98, *p* = .002, *η*^2^ = .333) were significant, as was the Task-Interference interaction (*F*_1,24_ = 20.87, *p* < .001, *η*^2^ = .465). For pairwise comparisons, we found no significant difference between high and low interference in the report average task (mean difference = 7 ms, *t*_24_ = 0.27, *p* = .790), but there was a significant difference in the report single task (mean difference = 205 ms, *t*_24_ = 4.46, *p* < .001, *d* = 0.89).

## Discussion

Our novel ensemble interference paradigm was able to replicate the well-established global precedence effect (Navon, 1977). Specifically, participants were faster and more accurate when reporting the global average orientation compared with reporting the local orientation of a single item from the set. This demonstrates the validity of our stimuli and experimental design for the investigation of global and local processing in ensemble encoding. Importantly, a novel result of Experiment 1 is the existence of an implicit local interference effect when extracting a global summary statistic from an ensemble, evident by reduced accuracy when reporting average orientation under high interference where the distractor was a single item from the set, compared with low interference where the distractor value was outside the set. This was not coupled with RT differences across the interference conditions, indicating this effect was not the result of a tradeoff between speed and accuracy.

Similarly, we observed a global interference effect for accuracy, where participants performed worse when reporting single orientation under high interference, compared with low interference. Reports of single orientation under high interference were significantly below chance, indicating that participants were more likely to select the mean value as the target, compared with the correct single item value. This is strong evidence of global interference, and is consistent with previous results showing the influence of the ensemble mean on single-item processing (Brady & Alvarez, 2011; Sama et al., 2019). Together, these results appear to demonstrate reciprocal interference between global and local processing in ensemble perception. The magnitude of interference at the global and local levels appears to be equal, since the relative decrease in accuracy from low to high interference was similar for reports of both average and single orientation, as evidenced by the non-significant Task-by-Interference interaction for accuracy. To our knowledge, this is the first demonstration of implicit local interference on global statistical processing in ensemble perception (but see Experiments 3 and 4, which question this conclusion).

The global interference effect predicts a slower RT when reporting single-item values during high interference. Surprisingly, we observed the opposite effect, where RT was faster when reporting single orientation under high interference compared with low interference. One possible explanation for this finding is that during low interference, participants may have adopted a decision strategy to reject the obvious distractor. This would create an additional processing step where participants would first identify the nonmember, and then choose the other item as the correct target. We test this hypothesis in the next experiment.

## Experiment 2

Experiment 1 demonstrated that participants were slower at reporting the orientation of a single item under low compared with high interference, which was opposite to the expected result predicted by the global interference effect. Here we test whether this finding can be explained by the presence of an additional cognitive strategy taking place only during low interference report single trials. Specifically, we hypothesize that participants were slower in low-interference report single trials because they first identified the outlier (i.e., the distractor) that was not a member of the set, and then selected the other probe item as the correct target, instead of simply recognizing the correct target in the first step.

### Stimuli and procedures

The stimuli and procedures were identical to those reported in Experiment 1 and the *General methods* with the exception of one important change to report single low-interference trials. Namely, we set the distractor probe to be a non-target single item from the ensemble, instead of an item outside of the set as in Experiment 1. In other words, both the target and the distractor were single items from the ensemble, but only one was cued with a circle placed onscreen during the 2AFC task. This modification tests whether participants’ slower responses in the report single low-interference condition in Experiment 1 were due to first rejecting the outlier and then selecting the correct single item, instead of appropriately recognizing the target single item in the first place. If participants were indeed using the former strategy, then responses in the modified report single low-interference condition here should not differ from those in the report single high-interference condition.

### Results

**Fig. 3:**
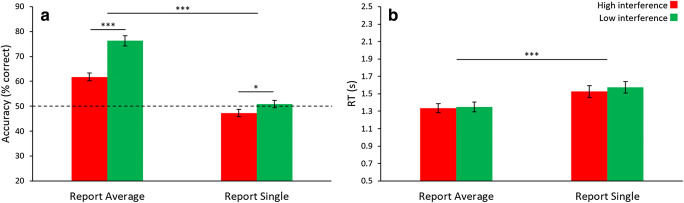
Results of Experiment 2. (**A**) Results from accuracy were similar to Experiment 1, namely the presence of a global precedence effect and reciprocal interference across global and local processing. Note that during report single low interference, participants were unable to discriminate between two single item probes (both of which were contained within the previously seen ensemble display, but only one of which was the correct item), as performance was at chance. (**B**) Reaction time (RT) shows a global precedence effect, but no interference effects. Error bars represent ± 1 standard error. * *p* < .05, *** *p* < .001

For accuracy (Fig. 3A), there was significance for Task (*F*_1,23_ = 91.24, *p* < .001, *η*^2^ = .799), Interference (*F*_1,23_ = 64.18, *p* < .001, *η*^2^ = .736), and the Task-Interference interaction (*F*_1,23_ = 31.29, *p* < .001, *η*^2^ = .576). Similar to Experiment 1, the difference between high and low interference was significant for reports of average (mean difference = 14.5%, *t*_23_ = 10.09, *p* < .001, *d* = 2.06) and single orientation (mean difference = 3.6%, *t*_23_ = 2.34, *p* = .028), and the performance in both interference conditions for reports of average orientation were significantly above chance (difference from chance > 11.8% for both report average conditions, both *t*s > 7.50, both *p*s < .001, both *d*s > 1.53). Unlike the previous experiment, performance in the report single interference conditions was not significantly different from chance (both differences from chance < 2.8%, both *t*s < 1.85, both *p*s > .155). Results for RT (Fig. 3B) revealed a significant main effect of Task (*F*_1,23_ = 42.63, *p* < .001, *η*^2^ = .650), but not Interference (*F*_1,23_ = 2.68, *p* = .115), and a non-significant Task-Interference interaction (*F*_1,23_ = 0.65, *p* = .428).

## Discussion

The present experiment successfully replicated the results of Experiment 1. Specifically, we observed the global precedence effect in both accuracy and RT and reciprocal interference effects were observed for accuracy. Regarding global interference, although performance in the report single high interference condition did not differ from chance, the significantly worse performance when reporting single orientation under high compared with low interference nevertheless reinforces the biasing influence of the mean on single-item processing that we have seen in the previous experiment.

Moreover, having the target and distractor both as items from the set when reporting single orientation under low interference eliminated the difference in RT between high- and low-interference conditions. Thus, consistent with our prediction, slower RT in the report single low-interference condition in Experiment 1 was likely due to participants first identifying and then rejecting the outlier, rather than correctly recognizing the target single item in the first place. Interestingly, this manipulation also had a pronounced effect on accuracy where performance did not differ from chance in the report single low-interference condition. This demonstrates that individual items from the set are not discriminable, and raises an interesting question: How can local interference be present in the absence of robust representations of single items? One possibility is that the precision of single-item representations varies along a continuum. At the lowest end of the continuum, representations are absent or imprecise, followed by some representation of single items without information about their spatial location, and finally at the high end of the continuum single-item representations are precise and bound with their spatial location. Representations of single items without awareness of their spatial locations would impair the ability to correctly assign one of two exemplar values to a cued location, and would thus explain the chance performance during the report single low-interference condition in the present experiment.

We explore the failure to remember the spatial location of individual items in greater detail in Experiments 4 and 5. But first, in Experiment 3 we evaluate the ability to judge the set membership of a candidate single item in the absence of both interference and cues to spatial location. This will lead to a more complete picture of how single items are represented in the ensemble.

## Experiment 3

Previous experiments explored single-item representation through an interference paradigm where participants reported the value of a cued single item from a specific location within the ensemble. In Experiment 3 we investigate the nature of single-item representation at a more elementary level, that is, in the absence of explicit interference and without the need to bind a stimulus feature to a spatial location. This will broaden our understanding of single-item representation in ensemble encoding and will serve as a foundation to explore the relationship between global and local ensemble processing in further experiments. Importantly, the results of Experiment 3 may provide insight into how local interference can apparently manifest in the absence of robust representations of single items. Finally, it is possible that the pattern of previous results in the high- and low-interference conditions is partially explained by an artefact of the testing procedure used. That is, high-interference distractors were more similar to the target, whereas low-interference distractors were more dissimilar to the target (with the exception of the report single low-interference condition in Experiment 2). To explore this possibility, in Experiment 3 we used a yes-no set-membership task where participants were presented with only a single probe item at test. If we still observe implicit biasing and interference effects using this paradigm, then target-distractor similarity at test cannot solely explain the pattern of results observed previously.

### Stimuli and procedures

The stimuli and procedures were very similar to those used in the previous two experiments, with two important modifications. First, we replaced the 2AFC interference paradigm with a set-membership identification task. Here, a single probe item was presented in the center of the screen following the ensemble display. The task was to judge whether or not it was a member of the previous set. Thus, this experiment focuses exclusively on reports of single orientation. Second, we removed the white circular location cue to allow reports of set membership that were independent of an item’s spatial location. Participants were instructed to press “1” if the item was a member of the previous set or “2” if the item was not a set member, thereby ensuring that a response was necessary for both possible decisions.

There were four possible stimulus conditions, categorized by the probe item’s assigned value. The probe item could be: (1) an actual randomly selected member of the set, (2) an item just outside the range of the set (i.e., one step, or 20°, outside the range, which was equivalent to the distance between items within the ensemble), (3) the mean orientation (which was never shown within the ensemble), or (4) an item far outside the range of the set (i.e., four to six steps, or 90–110°, outside the range). Like previous experiments, item values were spaced in 20° steps, and those that were closest to the mean were not selected as potential targets. Participants were blind to these manipulations. Data was labeled as percent “yes” responses for each condition, defined as the percentage of times participants labeled an item as a set member. We defined chance as the equal likelihood of accepting or rejecting a probe item as a set member, set at 50%. There were 360 total trials (15 predetermined means × 6 repetitions × 4 conditions).

### Results

**Fig. 4:**
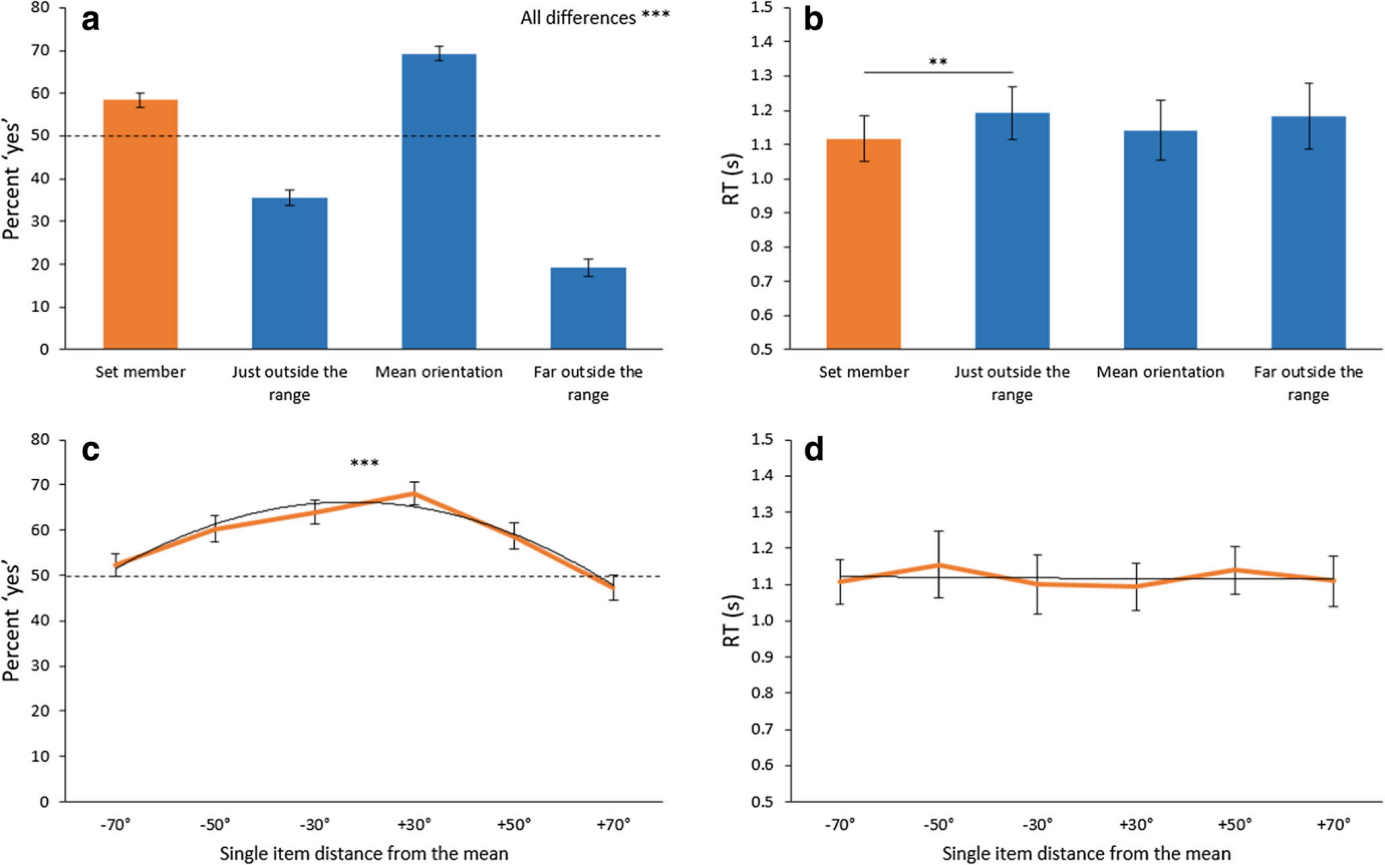
Results of Experiment 3. Responses to the actual set member are displayed in orange. (**A**) Results from accuracy show a strong bias to the mean, as the set mean was selected as a member more often than not, and more often than the actual set member. Results also show participants had a good representation of the range of the set, as items just outside the range were rejected more often than not, and items far outside the range were rejected more frequently than any other item. (**B**) Reaction time (RT) was roughly consistent across all trial types, demonstrating that performance was not a result of speed-accuracy-tradeoff. We also tested whether correctly labeling an item as a set member varied as a function of its distance to the mean (**C** and **D**). (**C**) For percent “yes” responses, there was a significant quadratic fit, where items closer to the mean had an increased likelihood of being selected. (**D**) For RT, there was no significant quadratic fit. Error bars represent ± 1 standard error. ** *p* < .01, *** *p* < .001

Percent “yes” responses and RTs were each analyzed using a single-factor within-subjects ANOVA comparing responses across the four different stimulus conditions. This was significant for percent “yes” (*F*_3,84_ = 218.63, *p* < .001, *η*^2^ = .886; Fig. 4A), as were all possible pairwise comparisons (all mean differences between 10.9% and 50.1%, all *t*s > 6.59, all Bonferroni *p*s < .001, all *d*s between 1.23 and 3.36). All conditions were also significantly different from chance (mean differences from chance between 14.4% and 30.8%, all *t*s > 4.61, all Bonferroni *p*s < .001, all *d*s between 0.86 and 2.66). The actual set member and the mean value were selected as set members more often than not (with the mean being selected significantly more often than the actual set member), and both items outside of the range were correctly rejected from set membership more often than they were labeled as set members (with the item far outside the range being rejected significantly more often than the item just outside of the range).

For RT (Fig. 4B), we found a marginally significant effect across the four conditions (*F*_1.9,54.2_ = 2.88, *p* = .066, *η*^2^ = .093), and pairwise comparisons revealed that this was driven by a significant difference between items that were set members versus items that were one step outside the range boundary (mean difference = 74 ms, *t*_28_ = 3.52, *p* = .008, *d* = 0.65). No other pairwise comparison was significant (all mean differences < 64 ms, all *t*s < 1.96, all *p*s > .360).

To further understand the representation of single items within the ensemble, we examined whether correctly labeling an item as a set member varied as a function of its distance to the mean. To that end, we organized the data from condition 1 (i.e., an actual member of the set) based on a probe item’s distance from the mean. Quadratic curve fitting was significant for percent “yes” (Fig. 4C; *F*_1,28_ = 60.19, *p* < .001, *η*^2^ = .682), but not for RT (Fig. 4D; *F*_1,28_ = 0.01, *p* = .958). Thus, probe items that were closer to the mean had a greater likelihood of being correctly selected as a set member, a bias that was not driven by differences in RT. Interestingly, performance for items at the range boundaries (±70° from the mean) was not significantly different from chance (both had < 2.5% difference from chance, both *t*s < 0.98, both *p*s > .999), but these items were selected as set members more often than the items one step outside of the boundary (*t*_28_ = 50.00, *p* < .001, *d* = 9.28). In contrast, performance for items within the ensemble range (±50° and ±30° from the mean) was significantly above chance (mean difference from chance = 12.8%, all *t*s > 2.88, all *p*s < .023, all *d*s between 0.53 and 1.33). We discuss the implications of this range effect next.

### Discussion

The results of Experiment 3 reveal three important findings with regard to global versus local representation in ensemble encoding. First, the mean of the ensemble implicitly biases the representation of single items. The mean was most likely to be selected as a member of the set despite it not being present in the ensemble display. Moreover, for actual set members, selection likelihood varied as a function of their distance to the mean. That is, items closer to the mean were correctly selected as set members more often. Second, participants have robust implicit knowledge of the range of the set. Specifically, single items within the range were correctly labeled as set members more often than not, performance for items at the range boundary was at chance, and items outside the range were rejected as set members more often than they were selected. Together with the previous point, this suggests that the effect of implicit knowledge of the range on ensemble encoding is relative as opposed to categorical. Finally, these implicit biasing and interference effects were observed using a task where only a single probe item was presented at test, arguing that the results of our previous experiments cannot be explained solely by confounds of target-distractor similarity in a 2AFC task. To further explore this issue, in Experiments 4, 5, and 6 we again use a 2AFC task but employ better controls for target-distractor similarity.

Given these findings, it is possible that the low accuracy for single reports under low interference in Experiment 2 was because the distractor was within the range of the set. Based on the results of Experiments 2 and 3, it appears that under high VWM load, participants do not have robust representations of single items from the set, but they do have a good representation of the range of the set. Thus, when reporting single orientation and both the target and the distractor are within the range of the set, participants will have difficulty discerning what the correct orientation value is. We explore a strong version of this prediction in Experiment 4. Taken together, these results demonstrate that the representation of ensemble range boundaries can be extracted implicitly and subsequently has strong effects on hierarchical ensemble encoding, which is consistent with recent findings (Hochstein et al., 2018; Huang, 2020; Khayat & Hochstein, 2018; Utochkin & Brady, 2020).

## Experiment 4

The previous experiment demonstrated that participants had a robust representation of ensemble range. In this experiment we investigate whether poor performance when reporting single orientation under low interference in Experiment 2 is due to implicit knowledge of the set range, as opposed to a failure to remember an orientation value at a specific spatial location. That is, when faced with a target and distractor that are both within the range of the set, participants will have difficulty discerning the correct orientation value. We test a strong version of this hypothesis that states that this should be true whether or not the distracting item was actually a member of the set (and thus would not occupy a spatial location in the previously seen ensemble). If knowledge of the range of the set is indeed interfering with reports of a single item’s orientation, then participants will not be able to correctly discriminate between a member and non-member of the set if both items are nevertheless within the range (regardless of whether or not they correctly remember the spatial location of the set member).

### Stimuli and procedures

The stimuli and procedures were identical to those used in Experiments 1 and 2, with one important difference, once again in the report single low-interference condition. Specifically, instead of being a non-member outside the range of the set (Experiment 1) or a member of the set (Experiment 2), the distractor was now a triangle that was within the range of the set, but was not actually presented within the previously seen ensemble. This distracting item was set to the middle value between two randomly chosen adjacent single items from the set (e.g., items 1 and 2, or 7 and 8). Importantly, the distracting item was never set to the middle value of the two items that were adjacent to the ensemble mean (i.e., items 4 and 5), since this value would be equal to the mean orientation itself (see Fig. 1A).

### Results

**Fig. 5:**
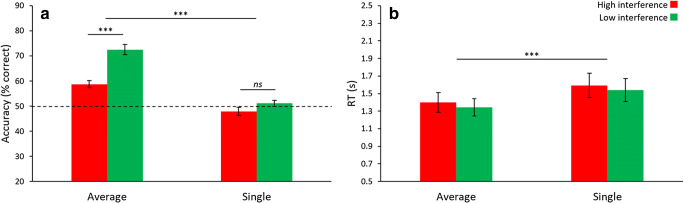
Results of Experiment 4. Both accuracy (**A**) and reaction time (RT; **B**) were consistent with the previous experiments’ results, particularly Experiment 2. Together, these results again show global precedence and interference, and importantly, that implicit knowledge of the range of the set interferes with reports of single orientation. That is, when reporting single orientation under low interference, participants are unable to disambiguate targeted single items from distractor single items when both items are within the range of the set (despite the distractor never being presented in the ensemble display). Error bars represent ±1 standard error. *** *p* < .001, *ns* = not significant (*p* > .05)

The repeated-measures ANOVA design was consistent with Experiments 1 and 2. For accuracy (Fig. 5A), we found significance for both Task (*F*_1,23_ = 69.83, *p* < .001, *η*^2^ = .752) and Interference (*F*_1,23_ = 48.11, *p* < .001, *η*^2^ = .677), as well as a significant Task-Interference interaction (*F*_1,23_ = 29.77, *p* < .001, *η*^2^ = .564). Pairwise comparisons found that the interaction was driven by a significant difference between high and low interference when reporting average orientation (mean difference = 13.8%, *t*_23_ = 9.34, *p* < .001, *d* = 1.77), but not when reporting single orientation (mean difference = 3.2%, *t*_23_ = 1.97, *p* = .061). Furthermore, we found that performance was significantly greater than chance when reporting average orientation under both high (difference from chance = 8.7%, *t*_23_ = 6.45, *p* < .001, *d* = 1.32) and low interference (difference from chance = 22.5%, *t*_23_ = 11.00, *p* < .001, *d* = 2.25). In contrast, performance in both report single orientation conditions was not significantly different from chance (both had < 2.14% difference from chance, both *t*s < 1.34, both *p*s > .385).

For RT (Fig. 5B), we found a significant effect of Task (*F*_1,23_ = 13.42, *p* < .001, *η*^2^ = .369), a marginally significant main effect of Interference (*F*_1,23_ = 3.37, *p* = .079), and a non-significant interaction (*F*_1,23_ = 0.04, *p* = .854).

Similar to the analysis in Experiment 3, we fit a quadratic curve comparing performance across all possible set members (i.e., the correct target in the 2AFC task) for reports of single orientation in both the high- and low-interference conditions. We also did this for all possible distractors in the report single low-interference condition (i.e., non-set members that were nevertheless within the range of the set). All three of these fits were not significant, both for accuracy (all *F*s < 2.50, all *p*s > .128 prior to Bonferroni correction) and for RT (all *F*s < 0.50, all *p*s > .486).

### Discussion

During single orientation reports, we predicted that target items that were set members would be indistinguishable from distractor items that were not set members, but were nevertheless within the range of the set. This was supported by the results of the present experiment. This effect held regardless of the relative distance probe values were from the mean. This is further evidence for the implicit extraction of the range in ensemble encoding. Together with the results of Experiment 3, which demonstrated that participants have good knowledge of the range of the set in the absence of requirements to remember the spatial location of single items, these results argue that interference in the report single task is better explained by implicit extraction of the range of the set (but we cannot conclusively rule out the influence of failing to remember the spatial location of individual items; future research should explore the relative weighting of these effects in greater detail). Interestingly, when reporting single orientation, the strength of the interference exerted by the mean and the range (i.e., an in-range item not from the set) were roughly equivalent, showing that global interference can manifest equally from two different summary statistics. It is also worth noting that we again replicated the global precedence effect in ensemble processing, which demonstrates the reliability and robustness of this effect.

In Experiment 3, we used a set-membership task and found that participants were more likely to select items as members based on their proximity to the mean (Fig. 4C), demonstrating the influence of global features on local processing. In this experiment, we returned to an interference-based 2AFC task and revealed additional evidence for the dominance of global perception. Namely, when reporting single orientation under high interference, participants were equally likely to select the mean over the correct single item regardless of that item’s distance from the mean. The same result was observed when the range served as the distracting item in the report single low-interference condition.

Finally, we again replicated the finding that participants were more accurate at reporting average orientation under conditions of low compared with high interference. We previously classified this as a local interference effect, but the results of the present experiment and Experiment 3 suggest that the interference is not likely attributed to local orientation values, since participants do not have robust representations of single items in our ensemble displays. Instead, we suggest these results are likely explained by interference from the range of the set, which would imply an interaction between different types of summary statistical processing in ensemble encoding. We test this prediction in Experiment 5.

## Experiment 5

The results of Experiment 4 demonstrated that participants implicitly encode the range of the display, and this representation of ensemble range interferes with the perception of single items. In Experiment 5, we investigate whether implicit extraction of ensemble range can also interfere with the processing of average orientation. Specifically, poorer performance in the report average high-interference condition in the previous experiments is likely attributed to the fact that the distractor was within the range of the set, and not necessarily because the distractor was a set member. Thus, we would expect to again see lower performance in the report average high-interference condition, compared with the low-interference condition, when the distractor is within the range of the set but was not actually presented within the previous ensemble display (i.e., the same manipulation we made in the report single low interference condition of Experiment 4).

### Stimuli and procedures

The stimuli and procedures were identical to those used in Experiment 4, except for a modified report average high-interference condition. That is, the distractor in report average high interference was now an item in the range of the previously seen ensemble, but it did not take on a value of one of the actual set members. This distractor manipulation is shared with the report single low-interference condition that was used in Experiment 4 and is again used in the current experiment.

### Results

**Fig. 6:**
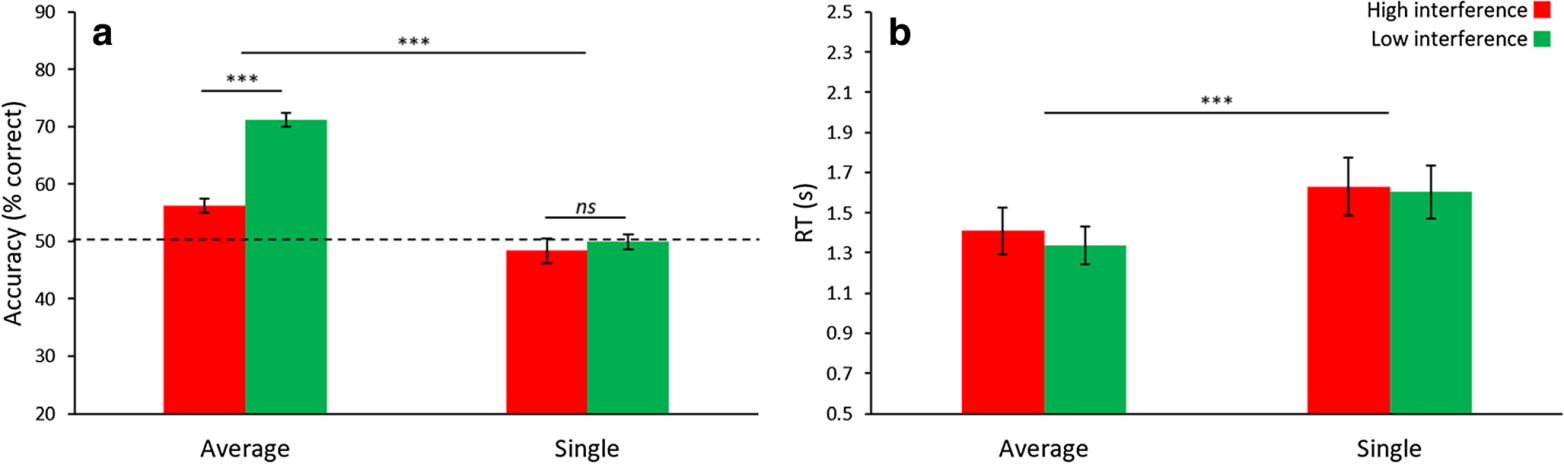
Results of Experiment 5, which completely replicated the findings of Experiment 4, both for accuracy (**A**) and for reaction time (RT; **B**). Error bars represent ± 1 standard error. *** *p* < .001, *ns* = not significant (*p* > .05)

For accuracy (Fig. 6A), the effects of Task (*F*_1,25_ = 64.33, *p* < .001, *η*^2^ = .720) and Interference (*F*_1,25_ = 30.47, *p* < .001, *η*^2^ = .549) were both significant, as was the Task-Interference interaction (*F*_1,25_ = 33.15, *p* < .001, *η*^2^ = .570). As was the case in Experiment 4, this interaction was driven by a significant difference in levels of interference for report average (mean difference = 14.9%, *t*_25_ = 8.09, *p* < .001, *d* = 1.53) but not report single (mean difference = 1.6%, *t*_25_ = 0.82, *p* = .427) tasks. Additionally, performance was significantly above chance when reporting average orientation under both high and low interference (both had > 6.3% difference from chance, both *t*s > 5.40, both *p*s < .001, both *d*s > 1.06). In contrast, the accuracy in both report single conditions was not significantly different from chance (both had < 1.6% difference from chance, both *t*s < 1.33, both *p*s > .389).

Like accuracy, the analysis of RT (Fig. 6B) also demonstrated a complete replication of the results of Experiment 4. Namely, the effect of Task was significant (*F*_1,25_ = 23.36, *p* < .001, *η*^2^ = .483), Interference was marginally significant (*F*_1,25_ = 3.51, *p* = .073), and the interaction was not significant (*F*_1,25_ = 1.37, *p* = .254).

As a further demonstration of the similarity in results across Experiments 4 and 5, independent sample *t*-tests comparing each of the four unique conditions across experiments returned non-significant results for both accuracy and RT prior to applying Bonferroni corrections (all *t*s < 1.39, all *p*s > .170, *df*s = 48).

## Discussion

The results of Experiment 5 were a complete replication of those in Experiment 4, even with the modified report average high-interference condition. Specifically, global interference from the ensemble mean and range on single-item processing were equal in magnitude (i.e., chance performance in both high- and low-interference conditions during report single, which did not differ), and we replicated the global precedence effect in ensemble processing (i.e., faster processing for average compared with single ensemble features) for the fourth time in this study. Importantly, we found the same poor performance in the report average high-interference condition when the distractor was within the range of the set, but was not actually presented within the ensemble display, compared with previous experiments where the distractor was a member of the set. This demonstrates that what we called a local interference effect in previous experiments is more accurately conceptualized as a range interference effect. Our findings that the summary statistics of ensemble range and mean interact are consistent with studies showing that altering the range of an ensemble can affect reports of the mean (Chong & Treisman, 2003; Maule & Franklin, 2015).

In contrast, Khvostov and Utochkin (2019) have suggested that the mean and the range of an ensemble are processed by independent cognitive mechanisms. To explain this discrepancy, we highlight some important methodological differences between our studies. First, the authors used a task requiring the explicit extraction of range, whereas in the present study range is encoded implicitly. Second, as noted by the authors and others (Hochstein et al., 2018), range can give some information about the mean, but the opposite is not true: the mean does not inform range estimates. To address this, Khvostov and Utochkin had participants match the range of a test set to a previously seen display set, but both ensembles contained different mean values. They showed that participants can still match the range between two sets containing different mean values, and thus argue for independent cognitive mechanisms mediating the extraction of both features. This does indeed show that the range of an ensemble can be extracted without using the mean, but we argue that it does not necessitate independence between the two (Maule & Franklin, 2015). In contrast, participants in our study encode the range of the set from the same ensemble display that they are making explicit judgments of mean orientation from, and we observe an interaction between the two summary statistics. In the final experiment of this study, we investigate the relationship between ensemble range and mean further by disentangling the numerical value of both metrics.

## Experiment 6

In all previous experiments, our ensemble stimuli were generated as a symmetrical uniform distribution, with equal distance between items. While some research has utilized asymmetrical distributions (Luo & Zhou, 2018), the majority of studies investigating ensemble encoding do not. Even when distributions are not uniform, they still tend to be symmetrical (e.g., Chong & Treisman, 2003). In a symmetrical uniform distribution, the mean and the center of the range are the same value. Given this, it is unclear whether participants are using an arithmetic mean or are simply centralizing the range boundaries when reporting an average ensemble feature (Hochstein et al., 2018). This raises the possibility that the range and mean arise from the same summary metric. Previous research has noted this problem when studying the range and mean of ensemble stimuli (Hochstein et al., 2018; Khvostov & Utochkin, 2019).

To resolve this ambiguity, in Experiment 6 we manipulated the ensemble distribution with the aim of disentangling the arithmetic mean from the center of the range, thus separating their potential influences. By doing so, we can investigate whether participants are using the arithmetic mean or the center of the range as their estimate of average orientation. Furthermore, investigating whether or not these summary metrics are distinct will shed light on the potential source of interference between the processing of ensemble mean and range observed in Experiment 5.

### Stimuli and procedures

To disentangle the mean and the center of the range, subsequently referred to as the centroid, we used asymmetrically skewed distributions. Individual triangles were -70°, -65°, -55°, -40°, -20°, 5°, 35°, and 70° from the centroid for a positively skewed distribution. Values in a negatively skewed distribution were -70°, -35°, -5°, 20°, 40°, 55°, 65°, and 70° from the centroid. This maintained a consistent range (140°), variance (50.5° in the present study compared with 49.0° in previous experiments), and set size (eight items) compared with all previous experiments. Thus, the main difference between this experiment and all previous experiments is in the use of an asymmetrically skewed distribution, which yields ensemble stimuli where the arithmetic mean and centroid have different numerical values. Figure 1C shows a positively skewed distribution. A negatively skewed distribution would be a mirror reversal. We generated ten preset ensemble centroid values. The mean was -17.5° from the centroid for positively skewed distributions, or 17.5° from the centroid for negatively skewed distributions.

Similar to previous experiments, we used a 2AFC task and asked participants to report average orientation in half of the trials and single orientation in the other half. These two tasks were randomly intermixed across trials. We utilized three distinct experimental conditions for each of the two tasks, defined by the specific pairing of 2AFC probe items. In one condition, both items occupied the same level on the ensemble processing hierarchy. That is, in the report average task the two targets were the arithmetic mean and the centroid (Mn vs. Cd; both are referred to as targets because either could serve as a correct response), and in the report single task the target and distractor were both single items from the set (Sn vs. Sn, with one being the cued target and the other being a distractor, similar to Experiment 2). In another condition one item was the centroid and the other was a single item from the set, with the single item serving as a distractor in the report average task (Cd vs. Sn) and the centroid serving as the distractor in the report single task (Sn vs. Cd). Finally, in the third condition one item was the arithmetic mean and the other was a single item from the set, with the distractor being the single item in report average (Mn vs. Sn) and the arithmetic mean in report single (Sn vs. Mn). Note that with this experimental design, all conditions are considered high interference, as both items in the 2AFC task contain values from within the ensemble set. Like previous experiments, participants were not informed about these manipulations, and were only cued to report the average or single orientation on each trial.

Items closest to the centroid and the mean were never selected as candidate single items. In previous experiments this corresponded to items 4 and 5 (see Fig. 1A). In the present experiment, these were items 5 and 6 for a positively skewed distribution, and items 3 and 4 for a negatively skewed distribution (see Fig. 1C).

The presentation and timing of ensemble stimuli on each trial were consistent with previous experiments. There were 720 trials (10 unique ensemble displays × 6 unique conditions × 2 skew directions × 6 repetitions). For data analysis, results from negatively skewed stimuli were transformed and combined with the positively skewed stimuli.

### Results

**Fig. 7:**
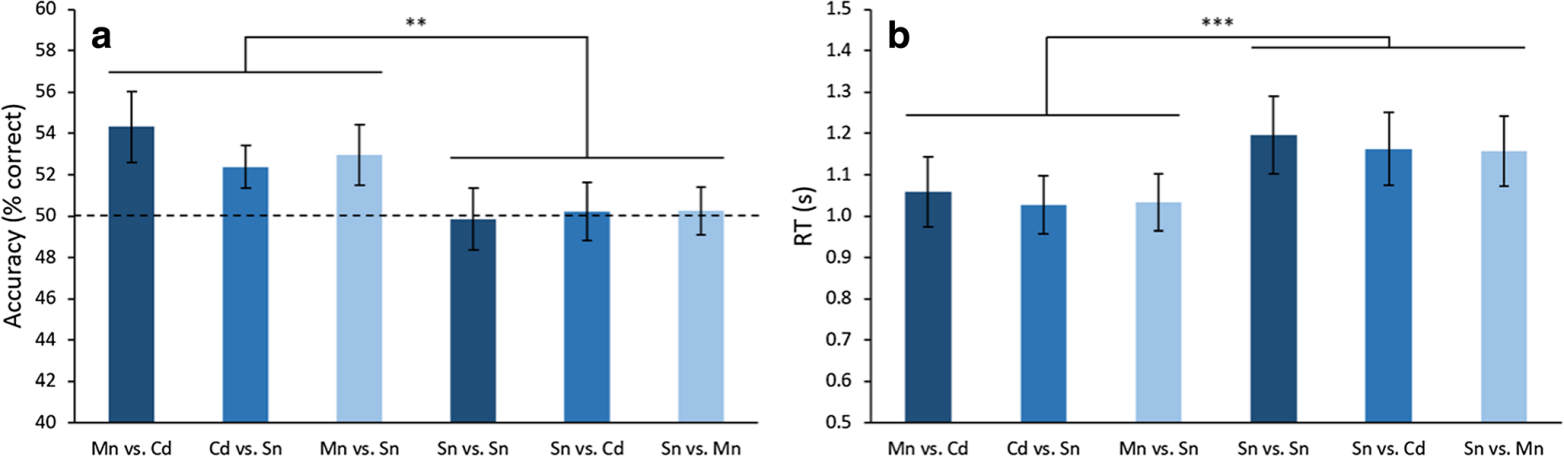
Results of Experiment 6, comparing (**A**) accuracy and (**B**) reaction time (RT) across all conditions. Conditions are labelled based on the target-distractor combination in the 2AFC task: mean (Mn), centroid (Cd), or single (Sn). For both accuracy and RT, we found differences between report average and report single, but not across the three different stimulus conditions, and no interaction. For report average, all accuracy values were significantly above chance, whereas all values were at chance for report single. Error bars represent ± 1 standard error. ** *p* < .01, *** *p* < .001

Accuracy and RT were both analyzed with a 2 (Task: report average or report single) × 3 (Condition: both 2AFC items from the same level on the ensemble processing hierarchy, Cd and Sn, or Mn and Sn) within-subjects ANOVA. For accuracy (Fig. 7A), we observed a significant effect of Task (*F*_1,23_ = 10.59, *p* = .003, *η*^2^ = .876), but a non-significant effect of Condition (*F*_2,46_ = 0.34, *p* = .713) and a non-significant interaction (*F*_2,46_ = 1.02, *p* = .368). Next, we found that performance in all three report-average conditions was significantly above chance (mean difference from chance = 3.2%, all *t*s > 3.20, all Bonferroni *p*s < .012, all *d*s between 0.65 and 0.72), while performance in all report-single conditions was not significantly different from chance (mean difference from chance = 0.1%, all *t*s < 0.28, all *p*s > .999). We observed similar results with RT (Fig. 7B). Namely, there was a significant effect of Task (*F*_1,23_ = 18.79, *p* < .001, *η*^2^ = .450), but a marginal effect of Condition (*F*_2,46_ = 3.05, *p* = .057), and a non-significant interaction between the two *F*_2,46_ = 0.06, *p* = .918).

**Fig. 8:**
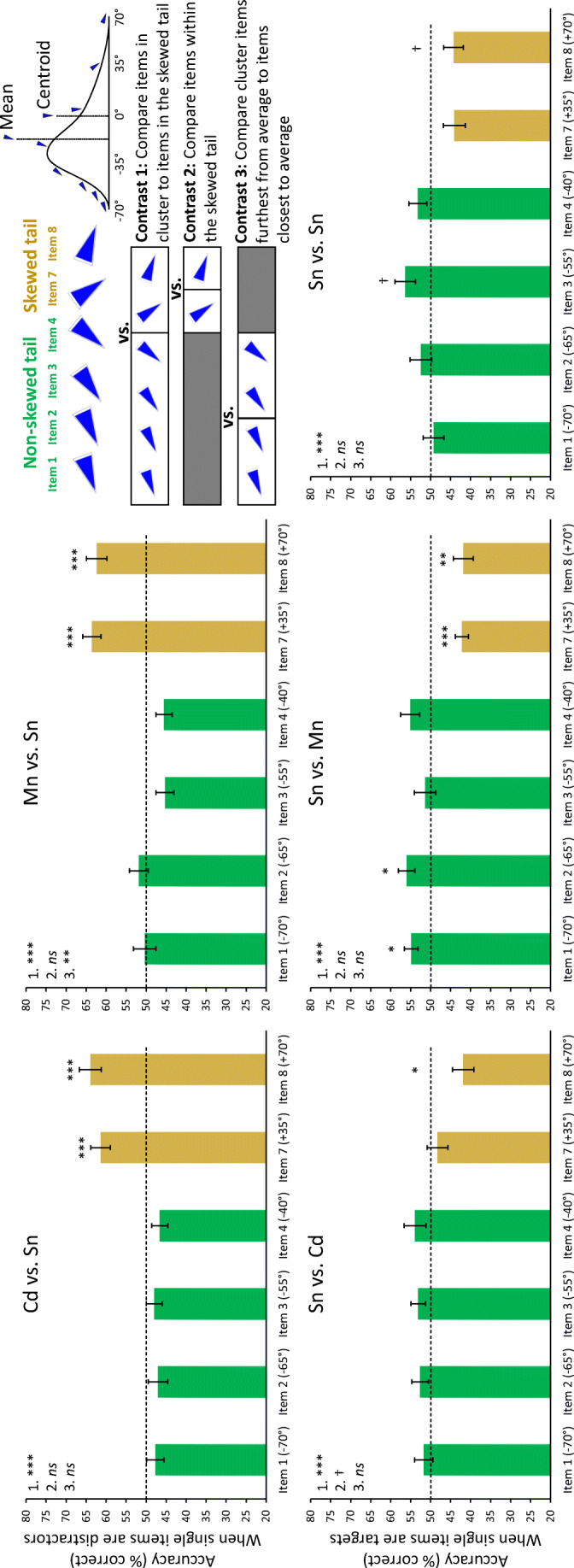
Evaluating the effects of single items in the skewed and non-skewed portions of the ensemble distribution. Each bar represents a specific single item. Green bars are items in the non-skewed portion of the ensemble distribution, whereas gold bars are items in the skewed tail. Single items were distractors during the report average task (top two graphs), and were targets during the report single task (bottom three graphs). Effects on accuracy were tested using three orthogonal linear contrasts (see top right for a pictorial illustration of each contrast). Contrast 1 compared accuracy for items in the non-skewed portion of the distribution (items 1–4) with items in the skewed tail (items 7 and 8). This was significant for all five conditions. Contrast 2 compared accuracy for the two items within the skewed tail (items 7 and 8). This was only marginally significant for Sn vs. Cd. Finally, Contrast 3 compared accuracy for items within the non-skewed portion of the distribution that were further from the arithmetic mean (items 1 and 2) with items that were closer to the mean (items 3 and 4). This was only significant for Mn vs. Sn. Asterisks above each bar indicate significance comparing accuracy against chance levels of performance (hashed line at 50%). Error bars represent ± 1 standard error. † *p* < .100, * *p* < .05, ** *p* < .01, *** *p* < .001

To further understand the impact of using an asymmetrically skewed distribution, we evaluated how the value held by single items, as either targets or distractors, and in both the skewed and non-skewed portions of the distribution, affected participants’ accuracy in both the report average and report single tasks. This was done for all conditions except for the arithmetic mean versus centroid (i.e., Mn vs. Cd), which did not contain single items. We were not able to employ quadratic curve fits, as the ensemble distribution was not symmetrical. Instead, we conducted three different linear orthogonal contrasts (illustrated in Fig. 8, top right, for a positively skewed distribution): (1) comparing items in the non-skewed portion of the distribution (Items 1–4) with items in the skewed tail (Items 7 and 8), (2) comparing the two items within the skewed tail (Items 7 and 8), and (3) comparing items within the non-skewed portion of the distribution that were further from the arithmetic mean (Items 1 and 2) with items that were closer to the mean (Items 3 and 4). For reports of average orientation (Fig. 8, top two graphs), the first contrast was significant for trials when the centroid was the target and also when the arithmetic average was the target (both *F*s > 49.51, both *p*s < .001, both *η*^2^ > .301). That is, accuracy when reporting average was higher when the distractor was a single item that came from the skewed tail compared with the non-skewed portion of the distribution. The second contrast was not significant for both report average conditions (both *F*s < 0.57, both *p*s > .120), showing similar accuracy when either item in the skewed tail served as a distractor. Finally, the third contrast was significant when the arithmetic average was the target (*F*_1,105_ = 5.55, *p* = .020, *η*^2^ = .046), but not when the centroid was the target (*F*_1,105_ = 0.01, *p* = .970). We discuss this finding in more detail in the *Discussion**.*

For reports of single orientation (Fig. 8, bottom three graphs), the first contrast was significant for all three conditions (all *F*s > 14.85, all *p*s < .001, all *η*^2^ > .116), indicating significantly poorer performance when the target was a single item from the skewed tail compared with the non-skewed portion of the distribution. The second contrast, comparing the two items in the skewed tail, was not significant for single versus single and single versus average (both *F*s < 0.01, both *p*s > .913), but was marginally significant for single versus centroid (*F*_1,105_ = 3.82, *p* = .053, *η*^2^ = .033). Finally, the last contrast – comparing items in the non-skewed portion of the distribution that were further away from versus closer to the arithmetic mean – was not significant for any of the three report single conditions (all *F*s < 2.46, all *p*s > .119).

When comparing performance to chance, we found significantly above-chance accuracy for both items in the skewed tail when the single item was the distractor (all *t*s > 4.63, all *p*s < .001, all *d*s > 0.94; Fig. 8, top two graphs), indicating that participants correctly rejected the single distractor more often than they selected it when it came from the skewed tail. When a single item from the skewed tail was the target, significantly below-chance accuracy was found for item 8 (the furthest item in the skewed tail) when either the centroid or the mean was the distractor (both *t*s > 3.08, both *p*s < .015, both *d*s > 0.63; Fig. 8, bottom two panes excluding Sn Vs. Sn). When item 7 in the skewed tail was the target, significantly below chance accuracy was observed when the distractor was the mean (*t*_23_ = 4.77, *p <* .001, *d* = 0.97) but not when it was the centroid (*t*_23_ = 0.66, *p* > .999). Finally, when items 1 and 2 from the non-skewed tail were targets, significantly above-chance accuracy was observed only when the mean was the distractor (both *t*s > 2.78, both *p*s < .032, both *d*s > 0.57; Fig. 8, bottom row, middle pane). All other comparisons were not significantly different from chance.

## Discussion

The results of this experiment, of Experiments 2–5, and of previous literature (e.g., Khayat & Hochstein, 2018) all demonstrate that participants are not only sensitive to the mean feature value of ensemble displays, but that they are also sensitive to the range. Furthermore, Khvostov and Utochkin (2019) suggest that the processing of ensemble mean and range are mediated by independent cognitive mechanisms, whereas the results of Experiment 5 in the present study suggest that they interact and are not independent. It is possible that we observed this interaction because the numerical value of the mean was equal to the center of the range (i.e., the centroid) in Experiment 5. In other words, it was not clear what participants’ computations of average orientation were based on, and the interference may have resulted from interactions between explicit (i.e., reports of average orientation) and implicit (i.e., items within the range boundaries) processing carried out by the same underlying cognitive resources, instead of from two distinct ensemble metrics. To examine this, and the processing of ensemble mean and range in greater detail, in Experiment 6 we disambiguated the numerical values of the arithmetic mean and centroid by using asymmetrically skewed distributions. We report three main findings.

First, participants were more sensitive to processing the arithmetic mean compared with the centroid of the distribution. When faced with a 2AFC task where either item could be the potential target, participants selected the arithmetic mean as the correct average orientation more often than they selected the centroid (Fig. 7, Mn vs. Cd). This is somewhat surprising, considering that the difference between the arithmetic average and the centroid was only 17.5°, which is smaller than the distance between single items on the uniform distribution in our previous experiments. Furthermore, participants may also have been drawn to the centroid because it was more numerically consistent than the average, which varied depending on the direction of skew. This did not occur.

Further evidence for increased sensitivity to the arithmetic mean came from the analysis comparing single items in the non-skewed portion of the distribution that were either further away from or closer to the arithmetic mean. Participants’ reports of average orientation were less accurate when the arithmetic average was the correct target and the distractor was an item closer to the mean, compared with items further away (Fig. 8, Mn vs. Sn, contrast 3). This was not observed when the centroid was the target (Fig. 8, Cd vs. Sn, contrast 3). This demonstrates that participants are likely more sensitive to the arithmetic mean compared with the centroid. This sensitivity is not likely explained by a proximity effect (i.e., interference when the distractor is in close physical proximity to the target), wherein the effect was observed with the arithmetic mean but not the centroid because the single items in question (i.e., items 3 and 4) are physically closer to the arithmetic mean on the orientation continuum. If a proximity effect were operating, then when reporting the orientation of single items, we should expect better performance for items 1 and 2 (i.e., the items furthest away from the mean in the non-skewed portion of the distribution) when the centroid was the distractor compared with the arithmetic mean, because these items are even further away from the centroid. However, we observed the opposite. Specifically, performance at reporting the orientation of items 1 and 2 was significantly above chance when the arithmetic mean was the distractor (Fig. 8, Sn vs. Mn), but was not when the centroid was the distractor (Fig. 8, Sn vs. Cd), despite the fact that items 1 and 2 are more proximal to the arithmetic mean.

Examining reports of single orientation for items 7 and 8 in the skewed tail of the distribution provided additional evidence for increased sensitivity to the arithmetic mean. When the centroid was the distractor, participants incorrectly chose it as the target more often than not only for item 8 (Fig. 8, Sn vs Cd, gold bars), but this occurred for both items when the arithmetic mean was the distractor (Fig. 8, Sn vs. Mn, gold bars). Note that these results are also in opposition to what would be expected based on a proximity effect, since items 7 and 8 are more proximal to the centroid, compared with the arithmetic mean. Taken together, all of these results suggest that participants are more sensitive to the arithmetic mean compared with the centroid, and thus when asking participants to compute average orientation, their reports are likely based on the former metric. These subtle differences would not be apparent in a symmetrical distribution and highlight the need for ensemble research to conduct more detailed investigations into differently shaped distributions.

Second, despite the increased sensitivity to the arithmetic mean compared with the centroid of an ensemble, the centroid can nevertheless serve as an adequate summary statistic in place of the arithmetic mean. For example, when reporting average orientation, accuracy was equivalent when either the centroid or the arithmetic mean was the target (Fig. 7, Cd vs. Sn and Mn vs. Sn). Similarly, both produce equivalent levels of interference on reports of single orientation (Fig. 7, Sn vs. Cd and Sn vs. Mn), which is consistent with the findings in Experiments 2, 4, and 5. Importantly, while the centroid is a sufficient summary statistic, our results show that it is nevertheless distinct from the arithmetic mean.

Third, items 7 and 8 from the tail of the skewed distribution were likely treated as outliers, but did not create a perceptual pop-out effect whereby their increased salience attracted attention and affected participants’ performance accordingly. In a symmetric uniform distribution used in previous experiments and other ensemble studies, the distances between items on one half of the distribution are equivalent to those on the other half. For skewed distributions in Experiment 6, it is possible that the items in the skewed tail of the distribution were perceptually distinct from the items in the non-skewed portion. In other words, it is possible that the skewed items were treated as outliers. Previous studies have demonstrated that outliers tend to be discounted during ensemble encoding (Haberman & Whitney, 2010; Hochstein et al., 2018). Our results are consistent with this finding. When the skewed items served as distractors in the report average task, performance improved (Fig. 8, Cd vs. Sn and Mn vs. Sn, gold bars). In contrast, when the skewed items served as the target in the report single task, performance suffered (Fig. 8, Sn vs. Cd and Sn vs. Mn, gold bars). This pattern of results would be expected if outliers were being discounted during ensemble encoding. These results further show that the skewed items were not creating a perceptual pop-out effect, in that if these items were more perceptually salient than the other single items, we would have expected superior performance in the report single task for items 7 and 8. Instead, performance for these items was at or significantly below chance (Fig. 8, bottom three graphs, gold bars). Finally, a consequence of discounting the skewed items as outliers is that the boundaries of the range are likely to have been modified. Support for this comes from the observation that performance when reporting average with the skewed items as distractors (Fig. 8, Cd vs Sn and Mn vs. Sn, gold bars) was comparable to the rejection rates for items one step outside of the range in the membership-identification task of Experiment 3. Thus, ensemble encoding is adaptive and efficient, since discounting outliers decreases the variability of a set and makes estimates of the mean potentially more reliable (Haberman & Whitney, 2010).

In conclusion, the results of Experiment 6 demonstrate that while the arithmetic average and centroid of an ensemble distribution share some similarities in ensemble encoding (e.g., their effect on single-item processing), they are nevertheless distinct perceptual constructs that do not share identical cognitive representations. Thus, the interference between the ensemble mean and range observed in Experiment 5 is not likely attributed to the possibility that the same underlying cognitive resource is used to represent both ensemble summary statistics. Instead, their representations may be distinct but overlapping, which could lead to independence in some experimental circumstances but interactivity in others.

## General discussion

The aim of this study was to elucidate the relationship between global and local feature processing in ensemble encoding. Supplementing our primary results is a successful replication of the global precedence effect five times, which speaks to the reliability and robustness of this effect and serves to validate our global-local ensemble interference paradigm. This is likely due to many shared mechanisms between ensemble encoding and other global-processing strategies, such as the statistical extraction of global spatial properties in scene perception (Harel et al., 2020; Malcolm, Groen, & Baker, 2016).

Our main goal was to provide a deeper investigation into the representation of local features in ensemble perception. To achieve this, we explored whether local features (i.e., the orientation of a single item from the set) could interfere with the processing of global features (i.e., mean orientation). In other words, we explored whether a local interference effect would manifest in ensemble processing, in addition to the expected global interference effect (i.e., interference from the mean when reporting the orientation of a single item). The results of Experiment 1 showed support for these hypotheses, revealing what appeared to be reciprocal interference between global and local ensemble processing. This was an interesting finding, given the multitude of studies that have demonstrated that detailed representations of single items are not necessary to form a representation of the ensemble average (for review, see Whitney & Yamanashi Leib, 2018). In the initial experiments of our study, it seemed possible that some single items were captured (Li, Castañón, & Solomon, 2017), which may have explained the local interference effect. However, the results of Experiment 2 demonstrated that participants did not have robust representations of single items from the set, and the results of Experiment 3 showed that participants instead had good implicit knowledge of another summary statistic, that is, the range of the ensemble stimuli.

These results led us to question how local interference can be observed in the absence of robust representations of single items. We reasoned that the robust representation of ensemble range boundaries observed in Experiment 3 could explain this apparent contradiction. In Experiments 4 and 5 we used distractors that were within the range of the set but were not actually presented in the ensemble and replicated the interference effects (both local and global) found in previous experiments. Thus, the previous instances of local interference were better characterized as a range interference effect, as the implicit processing of ensemble range interfered with the explicit processing of average orientation. In Experiment 6 we investigated if the presence of this interference was due to the fact that both summary statistics were derived from the same source, that is, the centroid or center of the range. In the symmetrical distributions used in all of our previous experiments, the centroid and the arithmetic mean were the same value. Thus, we disentangled the numerical values of the arithmetic mean and centroid by using asymmetrically skewed distributions in Experiment 6. We found that, when reporting average orientation, participants were more sensitive to the arithmetic mean, despite the relatively close proximity of the two values on the orientation continuum and the greater perceptual consistency of the centroid (i.e., the arithmetic mean changed value depending on the direction of skew, whereas the centroid did not). Thus, the interference observed between the ensemble mean and range may be explained by the presence of distinct, but possibly overlapping, perceptual and cognitive representations for each ensemble statistic.

Global-level interference on the processing of single items was replicated throughout the study, across numerous experimental manipulations, demonstrating that participants commonly perceived the mean as members of the set more often than the correct single items. This occurred despite the mean value not being present in any of the ensemble stimuli. Importantly, we are the first to demonstrate that this interference effect occurs with range too. That is, global interference on the representation of single items can come from two different summary statistics, and this interference appears equal in magnitude.

These findings are an important contribution to our understanding of global-local relationships in ensemble processing. Rather than manipulating the saliency of particular single items within the ensemble, we constructed a novel ensemble paradigm that varied the level of global-local interference. We found that even under high interference, the visual system does not capture, and is not influenced by, single item values when constructing ensemble representations. Instead, putative local interference effects are better explained by an interaction between the processing of ensemble range and mean values. Knowledge of the range boundaries is certainly beneficial when deciding if a single item is not a member of an ensemble, as they allow participants to accurately reject items that are outside the set range (Experiment 3; Hochstein et al., 2018). However, the tradeoff is poor accuracy when deciding if a single item is a set member, as any value within the range is likely to be selected (even if it is not a set member), with increased probability of being selected the closer the item is to the mean (Experiment 3). Thus, the mean and the range of an ensemble inform probabilistic estimates of set membership. This conforms with recent research comparing visual perception to Bayesian-like inference (Cashdollar, Ruhnau, Weisz, & Hasson, 2017; Fiser, Berkes, Orbán, & Lengye, 2010; Kersten & Yuille, 2003; Kording, 2014; Purves, Monson, Sundararajan, & Wojtach, 2014; Purves, Morgenstern, & Wojtach, 2015). These inferences make use of statistical information, commonly present in ensembles (Orhan & Jacobs, 2014). Comparing our findings to these models, a candidate single item acts as a marginal, and bias towards the mean is captured as a likelihood estimate, as the closer the items are to the mean, the more likely that they are perceived as a set member.

In mathematics, given a set of distribution parameters such as mean, variance, and skew, we can statistically infer the likelihood of a particular value coming from that distribution. This can be done without any knowledge of actual values that belong to that distribution. Alternatively, distribution parameters can also be calculated to fit a preselected set of values. It appears that the visual system operates based on the former principle, evaluating set membership based on how an object fits perceived distribution parameters, and not based on some stored representation of single objects. By extension, these perceived distribution parameters (e.g., summary statistics) are also not formed based on detailed single-object representations (Banno & Saiki, 2012; Haberman & Whitney, 2011; Lau & Brady, 2018; Ward et al., 2016). We would argue that bias from the mean and range occurs because these summary statistics serve as the most representative values of the set.

This interpretation reveals an apparent circular paradox in ensemble perception. That is, if set membership decisions are evaluated based on their relationship to the mean and range, how can the visual system calculate summary statistics in the first place? It is possible that representations of single objects are used to derive summary statistics in some circumstances, such as when processing objects of expertise (Curby & Gauthier, 2007; Wong, Peterson, & Thompson, 2008), or objects whose encoding is subject to a higher limit in VWM capacity (Curby, Glazek, & Gauthier, 2009). For lower-level orientation, which has a much more limited VWM capacity (Jiang, Shim, & Makovski, 2008), there are at least two possibilities.

First, Li et al. (2017) argued for robust averaging. This occurs when nonlinear transformations favor items closer to the mean, and the visual system preferentially selects these when calculating summary statistics. This appears to be counterintuitive, as some observations are inevitably discarded which would reduce efficiency. However, the authors showed that in situations where the system has noise (e.g., neural noise in visual cortex), use of robust averaging results in better accuracy compared to weighing each item equally. Based on our results, even if select items are used in robust averaging, they are immediately lost after constructing ensemble statistics.

While data from Experiment 3 mirrors the robust averaging model described by Li et al. (2017), it does not confirm robust averaging. It is unclear how the visual system weighs items closer to the mean when calculating ensemble statistics. Data from both the present study and Li et al. (2017) have not identified a plausible mechanism that can place greater weight on items closer to the mean despite not having robust representations of these single items.

The second possibility models ensemble encoding as a type of texture perception (Brady et al., 2017; Dakin & Watt, 1997; Im & Halberda, 2013; Morgan, Chubb, & Solomon, 2014a; Morgan, Raphael, Tibber, & Dakin, 2014b; Parkes, Lund, Angelucci, Solomon, & Morgan, 2001). Texture provides an estimate of variability, in that low variance ensembles will appear more homogenous than high variance ensembles (Lau & Brady, 2018). Given this, it is possible that some form of texture perception is mediating the extraction of ensemble statistical information such as average, variance, and range from sets of stimuli (Whitney & Yamanashi Leib, 2018). The data from Experiment 6 suggests skew as another important metric, but evidence that skew is extracted from texture information is mixed (Dakin & Watt, 1997; Motoyoshi, Nishida, Sharan, & Adelson, 2007). Nevertheless, there seems to be utility in considering ensemble encoding as a form of texture processing, a position supported by the considerable overlap between the image properties diagnostic of texture and ensemble perception (Portilla & Simoncelli, 2000). In light of this, it is perhaps unsurprising that similar underlying neural mechanisms have been implicated in the processing of textures and ensembles (Cant & Xu, 2012, 2017). Interestingly, a similar problem has been raised in research on numerosity, namely, how do participants quickly quantify the number of observed stimuli? Texture-processing models have achieved success in explaining this phenomenon (Morgan, Raphael, et al., 2014b). Taken together, these results suggest that appealing to mechanisms of texture perception may explain how summary information is extracted from large sets of objects, and further research may reveal how this is accomplished with little-to-no sensitivity to individual items from the set.

Alternatively, crowding effects may be responsible for limitations in participants’ abilities to extract the orientation of single items from the ensembles used in our study. Indeed, when items are clustered together outside of foveal vision, participants may have difficulty recalling the value of any one item (Whitney & Levi, 2011). However, crowding does not negate ensemble encoding (Parkes et al., 2001). Rather, the two cognitive phenomena may be complementary, in that crowding may confer an adaptive benefit to ensemble encoding. However, since it has been demonstrated that crowding and ensemble encoding may be governed by dissociable cognitive mechanisms (Bulakowski, Post, & Whitney, 2011), participants’ ability to report average orientation throughout our study is not likely explained solely by effects due to crowding. Instead, as mentioned above, the influence of crowding likely has a more pronounced effect on the processing of single items from the set. Future research is necessary to reveal the relative influence of global statistical processing and crowding on the representation of single items in a group of objects.

There are a number of unresolved issues in ensemble encoding that future work should investigate. First, how does attention affect the representation of single items, and does this affect the balance of global-local processing in ensemble encoding? Attentively targeting single items can bias summary reports (de Fockert & Marchant, 2008), but the question remains whether a single targeted item can be made salient enough to dissolve a summary representation altogether. For example, during a face-to-face conversation, would the visual system still extract crowd statistics, or is this irrelevant in these situations? A related question concerns how the processing of targeted single items within an ensemble compares to the processing of single items in isolation. This research is needed to more accurately model the continuum of object representations, from detailed perceptual capture of single items to the extraction of global statistical properties as the capacity limit of VWM is approached and ultimately exceeded.

Second, how do variations in set size affect the extraction of summary statistics such as the mean and range, and the nature of global-local processing in ensemble encoding? Research on smaller set sizes has shown that, when the set size is at or near the supposed VWM capacity limit of four items, it is still possible to capture detailed information about single items (Cowan, 2001; Luck & Vogel, 1997; Raffone & Wolters, 2001). Thus, true local interference may be observed for ensembles containing four items, and this may affect the formation of summary statistics. Conversely, less is known about the processing of ensembles with much larger set sizes. For example, are a majority of items integrated when forming a summary statistic of an ensemble containing 100 items, or is the statistic based on sub-sampling of a smaller group items (e.g., in parafoveal vision)? One study found that increasing set size up to 12 improved the accuracy of extracting average size and orientation (Robitaille & Harris, 2011), but further research should use much larger set sizes to examine when this improvement plateaus.

Third, what is the nature of single-item representation in higher-level stimuli? Some studies claim that the details of individual faces are extracted from face ensembles (Li et al., 2016; Neumann et al., 2018). However, the claim in these studies was based on single faces that were within the range of the set. Neither of these studies tested faces that were within range but were not set members. Our findings that items not present in the set but within the range boundaries are indistinguishable from actual set members calls into question the claim that features of single items can be extracted from brief ensemble displays. Instead, it is possible that these results are better explained by the implicit processing of the range of the face ensembles. Stronger evidence for single-item representations in ensemble encoding would entail showing that, in a set-membership task, single items within the range were chosen at a higher rate compared with single items that were within the range but were not members of the set.

Fourth, a more conservative evaluation of global precedence in ensemble processing would entail equating differences in accuracy across global (e.g., report average orientation) and local (e.g., report the orientation of a single set member) tasks. In such a case, would features at the global level still receive prioritized processing compared with local features?

Finally, people are able to build rich statistical representations of ensembles, extracting information not only about mean and variance, but distribution shape as well (Chetverikov, Campana, & Kristjánsson, 2016, 2017). While the extraction of mean and variance information from ensembles has been studied previously (e.g., Haberman et al., 2015; Solomon, 2010; Yang, Tokita, & Ishiguchi, 2018), the extraction of other statistics, such as kurtosis, has received far less attention (but see Atchley & Andersen, 1995; Dakin & Watt, 1997). If kurtosis is extracted, then we would expect to see, in a set-membership task, an exponential increase in selecting single items the closer they are to the mean value for leptokurtic ensembles, and the inverse for platykurtic ensembles. This may be due to the visual system modifying range boundaries to compensate for kurtosis, as we observed for skew in Experiment 6. However, bias to the mean may be a fairly common finding in ensemble encoding regardless of the shape of the distribution, as almost all ensemble studies which report such bias use uniform distributions, which essentially are extreme platykurtic distributions.

In conclusion, our findings increase our understanding of one of the more prevalent questions in ensemble processing, namely, the nature of the relationship between the processing of global statistics and local elements. We characterize what was previously described as interference from local features as more likely explained by an interaction between the representations of the mean and range of an ensemble. We also show that participants’ reports of average orientation are closer to the arithmetic mean then they are to the center of the range (i.e., the centroid), and that these ensemble metrics are perceptually distinct. Importantly, we suggest that the processing of ensemble mean and range are likely mediated by separable but overlapping and interacting cognitive mechanisms. Additionally, our study adds to the body of evidence that detailed features of single items from ensembles are not extracted. Instead, we show that judgments of local ensemble details are based on probabilistic inferences of set summary statistics, such as the mean and range. We therefore suggest that future studies of single-item representation in ensemble encoding should investigate the biasing influence of both the mean and range on the perception and recognition of single items from the set.

## Footnotes

1 We use the term “interference” in our study because of how we manipulated the different levels of the distractors in the 2AFC task used throughout the experiments, and to be consistent with the language that Navon (1977) used in his study, which inspired the design of our own. We could also characterize this as an “influence” of global over local processing, or a “bias” from global statistics on blurred representations of single items, but instead use “interference” throughout for the reasons outlined above.
